# Endothelial Nitric Oxide Production and Antioxidant Response in Breath-Hold Diving: Genetic Predisposition or Environment Related?

**DOI:** 10.3389/fphys.2021.692204

**Published:** 2021-07-09

**Authors:** Danilo Cialoni, Andrea Brizzolari, Michele Samaja, Gerardo Bosco, Matteo Paganini, Nicola Sponsiello, Valentina Lancellotti, Alessandro Marroni

**Affiliations:** ^1^Environmental Physiology and Medicine Laboratory, Department of Biomedical Sciences, Università degli Studi di Padova, Padua, Italy; ^2^DAN Europe Research Division, DAN Europe Foundation, Roseto degli Abruzzi, Italy; ^3^Apnea Academy Research, Padua, Italy; ^4^Department of Health Sciences, Università degli Studi di Milano, Milan, Italy; ^5^Cardiothoracic and Vascular Department, Azienda Ospedaliero-Universitaria Pisana, Pisa, Italy

**Keywords:** nitric oxide, oxidative stress, breath hold diving, genetic prone, diving

## Abstract

**Introduction:**

Nitric oxide (NO) is an essential signaling molecule modulating the endothelial adaptation during breath-hold diving (BH-diving). This study aimed to investigate changes in NO derivatives (NOx) and total antioxidant capacity (TAC), searching for correlations with different environmental and hyperbaric exposure.

**Materials and methods:**

Blood samples were obtained from 50 breath-hold divers (BH-divers) before, and 30 and 60 min after the end of training sessions performed both in a swimming pool or the sea. Samples were tested for NOx and TAC differences in different groups related to their hyperbaric exposure, experience, and additional genetic polymorphism.

**Results:**

We found statistically significant differences in NOx plasma concentration during the follow-up (decrease at T30 and increase at T60) compared with the pre-dive values. At T30, we found a significantly lower decrease of NOx in subjects with a higher diving experience, but no difference was detected between the swimming pool and Sea. No significant difference was found in TAC levels, as well as between NOx and TAC levels and the genetic variants.

**Conclusion:**

These data showed how NO consumption in BH-diving is significantly lower in the expert group, indicating a possible training-related adaptation process. Data confirm a significant NO use during BH-diving, compatible with the well-known BH-diving related circulatory adaptation suggesting that the reduction in NOx 30 min after diving can be ascribed to the lower NO availability in the first few minutes after the dives. Expert BH-divers suffered higher oxidative stress. A preliminary genetic investigation seems to indicate a less significant influence of genetic predisposition.

## Introduction

Nitric oxide (NO) is considered as one of the most important molecules regulating vascular adaptations during breath-hold diving (BH-diving; Lundberg et al., 2015) because of its action in the control of the cardiovascular system, blood flow, and blood pressure.

NO is involved in many physiological and pathological processes (Rand, 1992; Hou et al., 1999). Due to its short half-life (0.05–1.8 ms) (Liu et al., 1998), NO availability is ensured by a family of NO synthases (NOS), composed of at least three different isoforms (Moncada and Higgs, 1993).

The endothelial isoform (e-NOS) is mainly involved in modulating the vasodilator tone, vascular integrity preservation, and regulation of arterial blood pressure (Rand, 1992). eNOS also inhibits platelet aggregation and adhesion and enhances vascular permeability (Behrendt and Ganz, 2002). e-NOS levels are regulated by many factors, such as hypoxia and local substrate availability (Ostergaard et al., 2007), vascular shear stress (Chatterjee et al., 2008), and, most important, by different genetic variants (polymorphisms) (Ahsan et al., 2006; Wang et al., 2010). Indeed, functional variants in the endothelial NOS3 gene might alter the expression of the enzyme (Senthil et al., 2005).

Since NO is a radical, its levels are difficult to quantify (Liu et al., 1998), and it is preferable to measure stable NO derivates such as Nitrate and nitrites (NOx) (van Vliet et al., 1997) and byproducts of NO oxidation in blood and tissues (Moncada and Higgs, 1993). Specifically, NO is oxidated to nitrite (NO_2_) or, when oxyhemoglobin is available, to nitrate (NO_3_), with NO_3_ being predominant in blood circulation (Lundberg et al., 2008). Healthy individuals produce approximately 1 mmol of NO_3_ daily due to the oxidation of endogenously synthesized NO (Macallan et al., 1997). If necessary, NO_3_ can be reduced to NO_2_ by several enzymes, such as xanthine oxidase (Li et al., 2003) and xanthine oxidoreductase (Jansson et al., 2008). NO_2_ is further reduced to NO by different pathways, including hemoglobin (Cosby et al., 2003), myoglobin (Rassaf et al., 2007; Shiva et al., 2007a), xanthine oxidoreductase (Godber et al., 2000), and ascorbic acid (Carlsson et al., 2001). These pathways are significantly enhanced during hypoxia and acidosis to ensure NO production when the oxygen-dependent NOS enzyme activities are compromised (Giraldez et al., 1997; Ostergaard et al., 2007). In addition, NO_2_ reduction to NO during physiological hypoxia seems to contribute to physiological hypoxic signaling, vasodilation, and modulation of cellular respiration (Modin et al., 2001; Cosby et al., 2003; Shiva et al., 2007a,b).

Some studies have demonstrated an increase in circulating NO in breath-hold divers (BH-divers) after repetitive diving up to 20 m over 25 min in a pool and suggested possible correlations with physical exercise (Theunissen et al., 2013a). As BH-divers, marine mammals (Elsner et al., 1998) are subjected to post-diving ischemia-reperfusion when arterial and tissue oxygen levels are restored after reaching the surface, thus leading to an increase in reactive oxygen species (ROS) production (Dhaliwal et al., 1991; Bosco et al., 2010, 2020). Higher ROS levels can be harmful (Forkner et al., 2007; Diringer, 2008) by exacerbating the redox imbalance, increasing oxidative stress, and depleting acutely the antioxidant defenses of the body (Terraneo and Samaja, 2017).

Endothelial dysfunction has also been demonstrated in BH-diving (Brubakk et al., 2005; Obad et al., 2010) and can be explained by two hypotheses. First, the BH-diving-related transient hypoxia and accumulation of CO_2_ induce an increase in ROS levels, causing higher oxidative stress (Theunissen et al., 2013b; Mrakic-Sposta et al., 2019) and NO-related endothelial changes (Theunissen et al., 2013a). Second, the development of venous gas embolism, frequently observed in self-contained underwater breathing apparatus diving (SCUBA) despite correct decompression procedures (Valic et al., 2005), has also been recently demonstrated in BH-divers (Cialoni et al., 2016) and could play a role in the pathogenesis of BH-diving-related endothelial dysfunction.

Since NO is primarily released from arterial endothelium along with many other regulatory substances (Heitzer et al., 2001), any condition causing endothelial dysfunction inevitably affects NO levels and leads to cardiovascular diseases, such as coronary artery disease, peripheral arteriopathy (Drexler, 1997; Pepine, 1998), and atherosclerosis (Keaney and Vita, 1995; Tousoulis et al., 2010). Some studies have confirmed a primary role in increased oxidative stress caused by endothelium dysfunction in cardiovascular disease (Cai and Harrison, 2000). Thus, the investigation of hidden mechanisms predisposing BH-divers to the development of cardiovascular diseases is of paramount importance.

On the other hand, recent observations seem to indicate the existence of a genetic predisposition in developing BH-diving-related injuries (Cialoni et al., 2015) or diving reflex related adjustments (Baranova et al., 2017), but there is no clear evidence in the published literature whether NO availability and oxidative stress occurring in BH-diving are related to extreme environmental conditions (such as ambient pressure, salinity, and water temperature) rather than genetic predisposition. However, we also need to take into account that in niche sectors, such as diving, in which it is very difficult to plan genetic protocols in higher numbers of subjects, the recommendation in these conditions is to use biallelic markers [such as single nucleotide polymorphisms (SNPs)] to obtain indicative data even in lower numbers of subjects (Chandrika, 2001).

The study aims to investigate the changes in NO_x_ and antioxidant response [plasma total antioxidant capacity (TAC)] after a series of BH-dives in different environmental and hyperbaric exposure conditions. In the Supplementary Material, we also show the preliminary results related to the NO and oxidative stress response in BH-divers with different genetic variants of 10 selected polymorphisms.

## Materials and Methods

### Subjects and Dives

50 Expert healthy BH-Divers were studied in two settings: the first group during a series of deep dives at the swimming pool Y-40 “The Deep Joy^®^” (Montegrotto Terme, Italy) (42-m-deep); the second group during an open water training session at the Elba Island (Italy).

All the divers were informed about the risks and benefits of this study and read and signed a specific, informed consent form before the experiment. All the participants also signed a dedicated genetic informed consent allowing the genetic analysis. The study was conducted as per the Helsinki Declaration and was approved by the Ethical Committee of Università degli Studi di Milano, Italy (Aut. No. 37/17).

Subjects aged >18 years and non-pregnant women were included in the study. None of the subjects had previous or clinical evidence of arterial hypertension, cardio-pulmonary diseases, Taravana episodes (BH-diving-related loss of consciousness or seizure), or any other significant disease.

Subjects were asked to avoid food rich in NO_3_, such as red meat (Lundberg, 2009) and leafy green vegetables (Lundberg and Govoni, 2004). None of them took prescription drugs, suffered from any acute disease during the 15 days before the experiment, or reported assumption of anti-inflammatory medications, exposure to high altitude in the 7 days, or intense exercise during the 48 h before the investigation. None of the BH-divers performed any compressed-gas diving during the 30 days before the experiment.

All the subjects were affiliated to the “Apnea Academy” training agency as instructors or high-level divers; however, all the divers could easily reach a minimum of the following criteria:

– 20 m depth in constant weight;
– 3 min of static breath-hold (at the surface); and
– 75 m of dynamic BH-diving in a swimming pool (distance).

All the divers performed their usual training with a freely determined number and time of warm-up dives, bottom time, and surface intervals.

As per the “Apnea Academy” standard procedures, the training session involved a gradual approach to the maximum daily personal depth and an unrestricted number of deep dives, at the end of which all the divers returned to the laboratory for the post diving test protocol.

Diving profiles were recorded using a UP-X1 free-diving computer (Omersub S.p.a., Sovico, Italy), including mean depth, maximum depth (MD), and number of dives (ND). The free-diving computers measured and recorded data every 2 s. The included divers were stratified into several groups to be analyzed. First, divers were interviewed and divided by diving level (LD) in medium or high experience (ME vs. HE) considering their BH-diving skills, defined by years of activity, personal depth record, number of weekly training sessions, and certification level. Another stratification considered three parameters achieved on the day of the experiment, namely: average depth (AD), an average of MD reached, and an average ND; subjects were then divided into those who dived above (AD-above, MD-above, and ND-above) and those who dived below the calculated averages (AD-below, MD-below, and ND-below).

### Experimental Protocol

The protocol was the same in both the swimming pool and the sea tests.

Venous peripheral access was obtained from the antecubital vein of each subject, and blood samples were collected 30 min before the start of the diving series (basal). Blood samples were then collected 30 min (T30) and 60 min (T60) after the end of the BH-diving session, after discarding 5 ml of the blood to remove any clots. Plasma was obtained by centrifugation (3,000 rpm for 10 min) and was refrigerated at −20°C. Plasma samples were then delivered to the Laboratory of Biochemistry of the Department of Health Sciences (DISS) of the Università degli Studi di Milano for analysis.

Epithelial oral cells were also obtained using two buccal swabs from each volunteer. DNA was isolated using the ChargeSwitch kit (Invitrogen Corp., Carlsbad, CA, United States), following the instructions of the manufacturer, and both buccal swabs were suspended in 100 μl of elution buffer.

We investigated for the following:

✓ Differences in plasma concentration of NOx and TAC for the following diving risk factors:

• BH-LD (ME vs. HE)
• Environmental (swimming pool vs. sea)

✓ Differences in plasma concentration of NOx and TAC in the following recorded diving risk factors, between those above and those below the calculated average:

• AD (AD-above vs. AD-below)
• MD (MD-above vs. MD-below)
• ND (ND-above vs. ND below)

✓ Differences in plasma concentration of NOx and TAC (before and after the dives) in genetic variants of 10 investigated polymorphisms (as explained in the following sections).

### Plasma NOx Measurement

All 50 subjects were investigated for the NOx plasma level repeated for the three times specified in the protocol. Before the analysis, plasma was deproteinized. Briefly, 400 μl of the sample was treated with 400 μl of acetonitrile (Romitelli et al., 2007) to precipitate the proteins and centrifuged at 12,000 rpm for 10 min. NOx was measured in the deproteinized plasma using a method based on Griess’s reaction as an index of NO concentration (Green et al., 1982), according to Cialoni et al. (2019). Plasma NOx levels were obtained by interpolation of standard NaNO_3_ curves (Tsikas, 2005). All the samples were analyzed two times. Results were expressed as a percentage of difference of the control value (basal).

### Plasma TAC

A total of 38 subjects out of 50 were also investigated for TAC, using the ferric reducing antioxidant capacity (FRAP) assay (Benzie and Strain, 1996), with some modifications. Briefly, 45 μl of plasma was added to 1.5 ml of freshly prepared FRAP reactive in plastic tubes. After 5 min of incubation at 37°C, absorbance was read at 593 nm in a Uvikon 931 UV-VIS spectrophotometer (Northstar Scientific, Bardsey, United Kingdom). Aqueous solutions of FeSO_4_ 7H_2_O (100–1,000 μM) were used for the calibration curve. TAC values were obtained by interpolation of the FeSO_4_ calibration curve. All the samples were analyzed two times, and the results were expressed as FRAP value [μM Fe (II)] of the samples (Zarban et al., 2009).

### DNA Polymorphisms

About, 10 different genetic polymorphisms related to the investigated risk factors (available NO and oxidative stress) were analyzed, especially, 2 involved with NO availability, 4 with the anti-inflammatory activity, and 3 with the antioxidant capacity. Also, angiotensin-converting enzyme polymorphisms were analyzed.

The polymorphisms were analyzed using a real-time PCR (RT-PCR) technique. Specific primers and probes for the SNP rs1799983 were designed according to the TaqMan genotyping assay (Applied Biosystems, Foster City, CA, United States), while SNP rs2070744 was analyzed using primers and probes designed according to the Kaspar genotype assay (KBIoscience [B]). Both SNPs were analyzed on ABI 7900 following the instructions of the manufacturer.

In all the investigated polymorphisms, the NOx and TAC levels before the diving and at follow-up (T30 and T60) for the different genetic variants were analyzed.

### Statistical Analysis

Data are presented as mean and SD for parametric data and median and range for non-parametric data. To minimize the subject-to-subject variability, data were normalized against the basal value. The Shapiro–Wilk normality test was used to verify a Gaussian distribution. Genetic data were compared using a one-way ANOVA for multiple comparisons or the Friedman test for multiple comparisons for parametric and non-parametric data, respectively. NOx and TAC were compared using a Mann-Whitney test or an unpaired *t*-test.

A probability lower than 5% was assumed as the threshold to reject the null hypothesis (*p* < 0.05).

## Results

A total of 50 healthy BH-Divers (40 male and 10 female; mean age 43.24 ± 9.8; mean height 176.3 cm ± 7.1; mean weight 74.4 kg ± 10.4; and BMI 23.8 ± 2.4) were studied in two different environmental conditions: 22 at the swimming pool and 28 at sea (Supplementary Table 1A).

The overall diving profiles showed an AD of 22.2 ± 8.5 m, *n* = 23 AD-above, and *n* = 27 AD-below; MD of 33.2 ± 8.2 m, *n* = 24 AM-above, and *n* = 26 AM-below; and an ND of 16.5 ± 5.8, (*n* = 26 ND-above vs. *n* = 24 ND-below) (Supplementary Table 1B).

Subjects were also classified as HE of *n* = 28 and ME of *n* = 22 (Supplementary Table 1A).

The groups obtained by dividing the sample in above and below the average (AD, MD, ND-above vs. AD, MD, ND-below, respectively) showed significant differences between the more performant subjects as compared with the less performing ones in terms of AD(AD-above vs. AD-below); MD (MD-above vs. MD-below) and ND (ND-above vs. ND-below) confirming a different diving exposure in the two groups (above vs. below).

Similar significant differences were also found between more experienced and less experienced divers [ME vs. HE and (Supplementary Table 1B)].

We did not find any statistical differences in BMI and age in the groups selected (Supplementary Table 1C) as the MD and ND were not statistically different in the two investigated environments (swimming pool vs. sea). We only found a higher mean of depth in the swimming pool group as compared with the sea group (Supplementary Table 1C).

**FIGURE 1:**
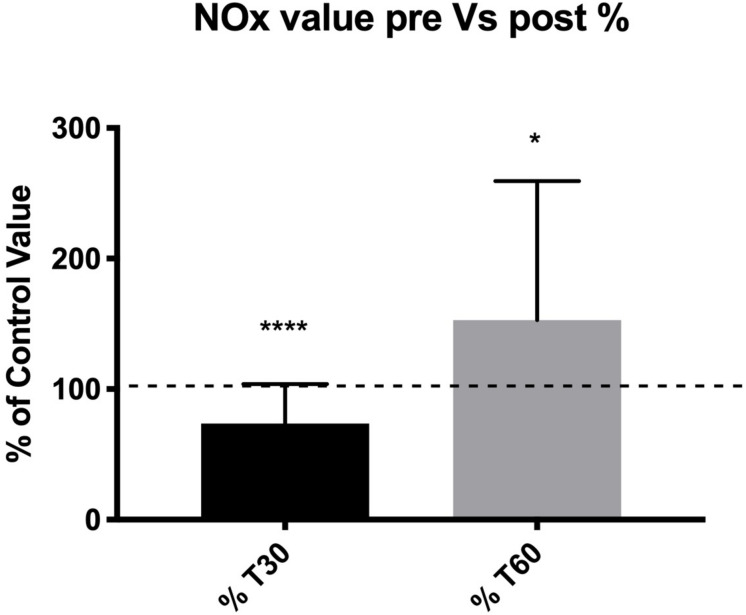
Subjects showed a lower NOx concentration at T30 suggesting a lower NO availability after dives, and a higher NOx concentration at T60 suggesting a NO availability re-balance effect. *p < 0.05, ***p < 0.001.

Regarding overall plasma NOx concentration, a significant decrease of –27.6% at T30 (73.5% of the control value, *p* < 0.0001) and a significant increase of +24.1 at T60 (124.1%, of the control value, *p* < 0.012) were found. All these differences (decrease at T30 and increase at T60) were statistically significant in terms of the percent of the pre-diving control value (Figure 1).

**FIGURE 2:**
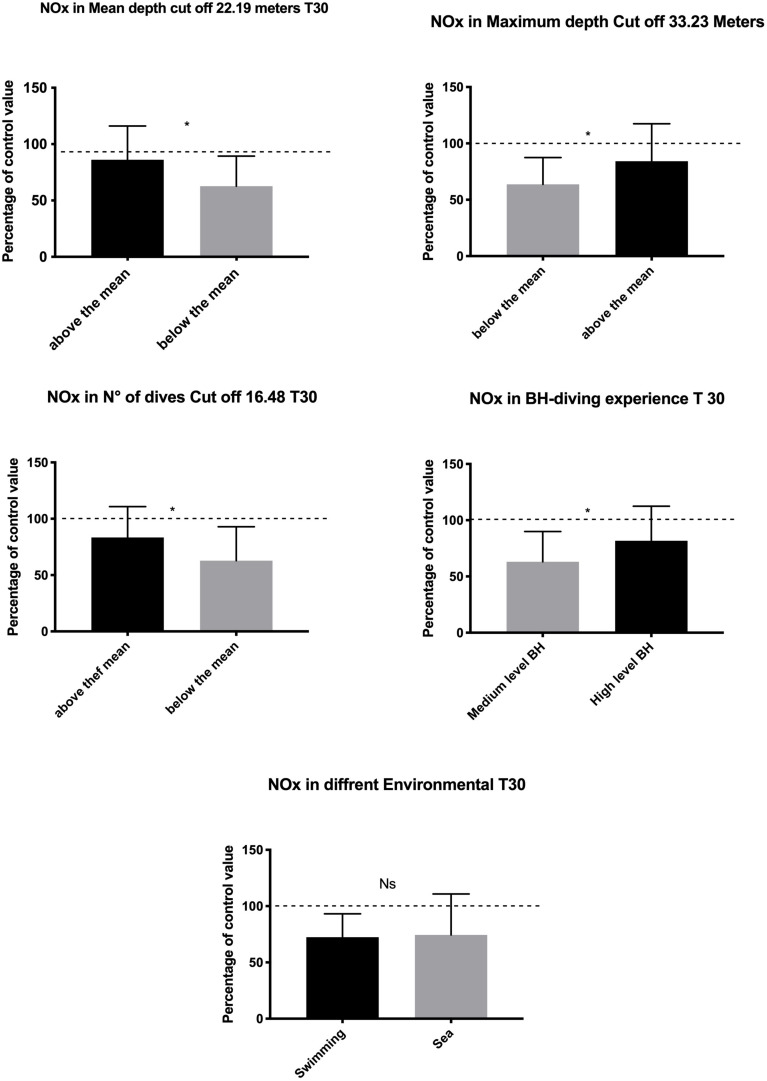
Data at T30 show lower NO usage in highly exposed and experienced subjects indicating adaptation in the management of hyperbaric-related vascular changes. Swimming Pool vs. sea BH-diving data showed no significant differences. *p < 0.05.

Regarding the differences in blood NOx concentration among the groups, a significantly lower decrease was found at T30 in experts (AD, *p* = 0.002; MD, *p* = 0.01; ND, *p* = 0.01; DL, *p* = 0.03) (Figure 2).

The difference, if any, in NOx plasma concentration was detected comparing the swimming pool vs. the sea setting (*p* = 0.81) (Figure 2).

**FIGURE 3:**
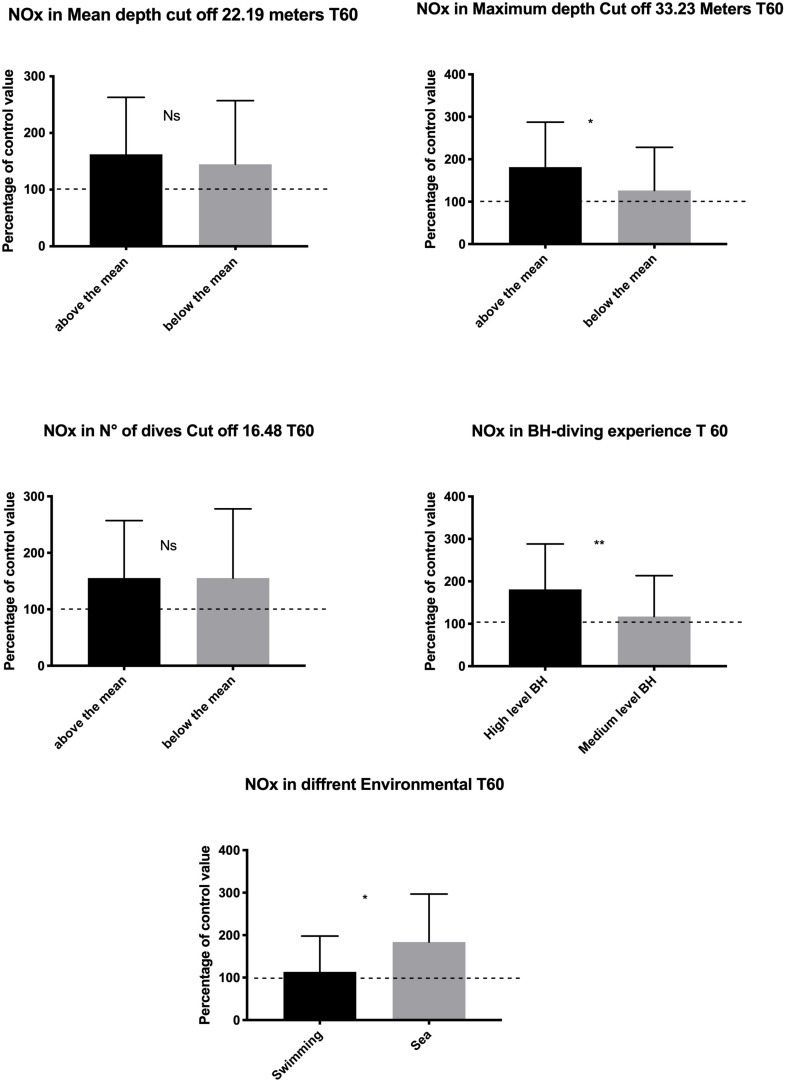
Data at T60 show a rebound effect with an increase of NO available probably to compensate for the higher underwater consumption. This rebound effect was larger in the subject who shows a higher diving exposure and experience and in sea diving as compared with the swimming pool diving. *p < 0.05, **p < 0.01.

At T60, a higher increase of NOx value was found in subjects with higher diving exposure in terms of MD (*p* = 0.018) and LD (*p* = 0.006). In contrast, the mean depth and the ND were not associated with significant changes in NOx levels. Finally, a higher NOx increase at T60 was found in the sea group than in the swimming pool group (*p* = 0.014) (Figure 3).

**FIGURE 4:**
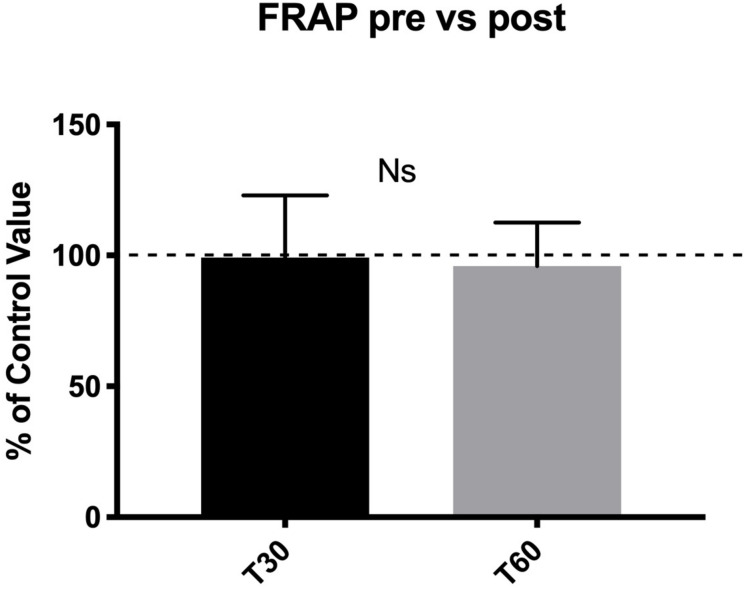
No statistical difference in TAC levels were found between pre- and post-dive values at T30 and T60.

No statistical difference was detected in TAC levels between pre- and post-dive values (Figure 4).

Similarly, no differences were found at T30 in the groups above vs. below average for the four-diving risk levels (AD, MD, ND, and LD) and in the two different environmental conditions (sea vs. swimming pool). Only the follow-up at T60 demonstrated higher oxidative stress levels in divers who performed BH-diving above the mean of MD and in HE divers HE (*p* = 0.01 and *p* = 0.03, respectively).

No significant difference was found as well between the swimming pool and the sea BH-dives.

Finally, no significant relationship was found in the NOx and TAC levels in the different genetic variant (Supplementary Table 2).

## Discussion

The NOx (Tsukiyama et al., 2017) and the TAC changes (Marciniak et al., 2009) after a BH-diving training session were investigated in a group of 50 BH-divers divided into two groups with different hyperbaric exposure and different experiences.

In the appendix to this primary aim, we also observed the behavior of NOx and TAC in the same divers sorted by their genetic variant of 10 anti-inflammatory, vascular, and antioxidant related polymorphisms.

Nitric oxide (Koshland, 1992) plays an essential and complex role as a signaling molecule in several human physiological and pathological responses, including diving and hyperbaric-related adaptations (Theunissen et al., 2013a; Sureda et al., 2014). NO behavior is a complex molecule to be studied, for these multifaceted actions and the short half-life (Liu et al., 1998), especially, when adding the challenging test conditions of an extreme environment. A standard method to investigate plasma NO changes is by looking at the variations of its oxidation products, NOx, because the half-lives of NO_3_ and NO_2_ in blood circulation are 5–8 h and 20–40 min, respectively (Dejam et al., 2007; Lundberg et al., 2008). Physical exercise increases eNOS activity and resulting in a higher level of circulating NOx (Jungersten et al., 1997; Green et al., 2004).

Under particular conditions, such as hypoxia, NOx can be reconverted to NO through different pathways involving proteins (hemoglobin and myoglobin), enzymes (xanthine oxidase and xanthine oxidoreductase), and ascorbate to ensure NO production when O_2_ supply is reduced. In blood vessels, NO_2_ generates vasodilatory NO by reacting with deoxygenated hemoglobin (deoxy-Hb) and contributes to physiological hypoxic blood flow regulation. When hemoglobin O_2_-saturation drops to 50%, the reduction of NO_2_ to NO is enhanced. This effect results from two mechanisms: the availability of deoxyhemes (reaction substrate) to bind NO_2_, which is maximal in deoxygenated hemoglobin, and the amount of oxygenated hemoglobin tetramer, which increases the intrinsic reactivity of the heme with NO_2_ (Lundberg et al., 2008).

In a recent study, data obtained from an underwater blood draw (−42 m) carried out on 12 expert BH-divers clearly showed the NOx kinetics in BH-diving. These data indicate a significant underwater increase in plasma NOx concentration (+410.5% compared with pre-dive value) and an immediate return to baseline values after reaching the surface (Cialoni et al., 2021). These data confirmed a significant use of NO during BH-diving, compatible with the well-known BH-diving-related circulatory adaptations, but unexpectedly showed a swift return of circulating NOx to basal levels at the surface. This last aspect confirms that the NOx measured after diving reflects the availability of NO in real-time, without any diving-related “accumulation” effect in tissues. This observation helps to understand the results reported in this new protocol performed after a BH-diving training session, where we found a decrease of NOx 30 min after the training session, followed by an increase at T60. These data partially confirm a previous study where a similar increase was found, although without any initial decrease (Theunissen et al., 2013a). This difference could be easily explained by the different diving protocols of the previous test compared with that proposed in this research, especially concerning the ND and the descent technique.

Therefore, the T30 post-diving reduction of NO_x_ found in this experiment can be ascribed to the lower NO availability in the first few minutes after the dives caused by the higher use of this molecule during diving (Cialoni et al., 2021) to ensure the BH-diving related vascular adaptations. On the other hand, an increase at T60 could be a rebound of the efforts of the body to restore basal conditions after exceptional stress exposure (Figure 1).

Hyperbaric exposure-related oxidative stress is the second aspect taken into account due to the consequences potentially affecting BH-divers. Indeed, BH-diving results in higher ROS production and oxidative stress, as confirmed by several authors (Theunissen et al., 2013a; Mrakic-Sposta et al., 2019; Cialoni et al., 2021), along with the activation of endogenous antioxidant defenses (Bulmer et al., 2008; Sureda et al., 2015). As recently suggested (Cialoni et al., 2021), oxidative stress is transitory, increasing in the underwater phases but returning near pre-dive levels after reaching the surface.

Unlike the previous paper (decrease of TAC: −60% to pre-dive value) (Cialoni et al., 2021), TAC did not show any difference between pre- and post-diving in the present experiment. This fact suggests the absence of an oxidative stimulus at the end of the training session, despite the hyperbaric exposure.

The data, in this study, indicate significant differences in NO consumption only when stratifying the divers into the two groups of high or low hyperbaric exposure or in the two groups of more expert vs. less expert subjects.

It is also intriguing to note that the lower NO consumption was observed in expert divers (HE) and those with higher hyperbaric exposure on the day of the experiment. We can hypothesize that a possible adaptation effect was undergone in these subjects who trained more intensely or had higher performances on the day of the investigation, with respect to the BH-divers that dived below the average. This aspect can also be explained by the “relax and comfort” training and diving techniques adopted by expert BH-divers, most likely decreasing the NO necessary to support the BH-diving induced hyperbaric-related physiological changes. However, this variable was not explicitly investigated and is worthy of further assessment in the future.

Another important observation concerns the changes in NOx and TAC when comparing swimming pool and sea exposures. Data at T30 were similar in the two different environments indicating a low influence of variables such as temperature (34 vs. 24°C) and salinity (freshwater vs. seawater). Therefore, NOx level changes are probably more influenced by the magnitude of hyperbaric exposure. An in-depth analysis of this aspect showed that the rebound effect noted at T60 is significantly higher in the sea subjects than in the swimming pool subjects (183.9 ± 112.9 vs. 113.5 ± 84.3). This could be related to the characteristics of sea BH-diving, requiring complex logistics for the training sessions, the use of a boat and a diving suit, more time inside the water (even if the ND is similar in the two groups), and more demanding environmental conditions (e.g., colder temperatures, waves, and currents).

However with all the limits that our genetic investigation shows, it is intriguing to note that the data did not show any differences in NOx and TAC values in the single nucleotide variant in all the 10 polymorphisms investigated. If this data will be confirmed by future studies, more focus on the genetic aspect could be indicated to confirm that the genetic predisposition is less critical as compared with hyperbaric exposure when concerning BH-diving related NOx and oxidative stress.

## Conclusion

This study showed the importance of hyperbaric exposure and expertise regarding NO availability and oxidative stress in BH-divers. NO consumption seemed to be significantly lower in high-performance BH-divers and the expert group indicating a possible training-related adaptation process. On the other hand, expert BH-divers demonstrated higher oxidative stress due to higher hyperbaric exposure in the sessions. A preliminary genetic investigation seems to indicate a lack of specific influence of genetic predisposition as compared with the increase of diving exposure.

## Data Availability Statement

The raw data supporting the conclusions of this article will be made available by the authors, without undue reservation.

## Ethics Statement

The studies involving human participants were reviewed and approved by Ethical Committee of Università degli Studi di Milano, Italy (Aut. No. 37/17). The patients/participants provided their written informed consent to participate in this study.

## Author Contributions

DC proposed the protocol and the search strategy, extracted and analyzed the data, and wrote the first draft. AB was involved in the conception and design of this work, reviewed the critical appraisal of selected articles, and assisted with the compilation of the systematic review. MP and NS extracted and analyzed the data and reviewed the manuscript. VL was involved in the test on the field and reviewed the manuscript. MS, AB, and AM supervised the entire process. All authors contributed to at least three of the four major components of a study and were involved in the conception and design of this work, contributed to the process of writing, and approval of the final manuscript.

## Conflict of Interest

The authors declare that the research was conducted in the absence of any commercial or financial relationships that could be construed as a potential conflict of interest.
