# Supplementary material for: Endothelial Nitric Oxide Production and Antioxidant Response in Breath-Hold Diving: Genetic Predisposition or Environment Related?
Source: Front Physiol. 2021 Jul 9;12:692204. doi: 10.3389/fphys.2021.692204 (PMC8300565; doi:10.3389/fphys.2021.692204)
Supplement: Supplementary file 1 [file Table_1.DOCX]

**Endothelial Nitric Oxide production and Antioxidant Response in BH Diving:**

**genetic predisposition or environment related?**

D. Cialoni^1,2,3^, A. Brizzolari^2,4^, M. Samaja^4^, G. Bosco^1^, M. Paganini^1^, M. Pieri^2^, N. Sponsiello^3,^ V. Lucchini^5^, V. Lancellotti^6^, and A. Marroni^2^

1) Environmental Physiology and Medicine Laboratory, Department of Biomedical Sciences,

University of Padua, Padua, Italy.

2) DAN Europe Research Division, Roseto degli Abruzzi, Italy

3) Apnea Academy Research, Padua, Italy

4) Department of Health Sciences, Università degli Studi di Milano, Milan, Italy

5) NGB Genetics Srl c/oUniversità di Ferrara - Bologna, Italy

6) Cardiothoracic and Vascular Department, Azienda Ospedaliero-Universitaria Pisana (AOUP), Pisa, Italy

Contact Information: Cialoni Danilo, DAN Europe, Contrada Padune 11, 64026 Roseto degli Abruzzi (TE), Italy. Phone: 39.085.8930333; Fax: 39.085.8930050; E-mail: dcialoni@daneurope.org

Keywords: Nitric oxide; Breath-Hold Diving; Diving; oxidative stress

Table I Typology and characteristics of study groups

A) Anthropometric data; and numbers of subjects into the different risk factor selected

B) Creation of two groups by different diving exposure/environmental/experience.

Lower hyperbaric exposure in the lower expert group is evident.

C) BMI and AGE: data were homogeneous in the different groups.

A)

| **Sample description N=50** |  |  |  |
| --- | --- | --- | --- |
| Gender |  | Female=10 (20%) | Male=40 (80%) |
| AGE |  | 43.2 | +/- 9.8 |
| Height |  | 176.3 cm | +/- 7.1 |
| Weight |  | 74.4 kg | +/- 10.4 |
| BMI |  | 23.8 | +/- 2.4 |
| **Diving description** |  | **Above the average** | **Below the average** |
| Average depth |  | AD-above= (31) | AD-below= (19) |
| Maximum depth |  | MD-above= (31) | MD-below= (19) |
| N° of dives |  | ND-above= (26) | ND-below= (24) |
| BH-Diving experience |  | HE= (28) | ME= (22) |
| Environmental |  | Sea= (28) | Swimming= (22) |

B)

| **Risk factors** | **Total mean** | **Above the mean** | **Vs** | **Below the mean** | **P=value** |
| --- | --- | --- | --- | --- | --- |
| Mean of depth | 22.2 +/- 8.5 | 27.2 +/-6.8 | Vs | 14.0 +/-3.1 | <0.0001 |
| Maximum depth | 33.2 +/- 8.2 | 38.5 +/-4.8 | Vs | 25.0 +/-5.3 | <0.0001 |
| N° of dives | 16.5 +/- 5.8 | 20.8 +/- 4.3 | Vs | 11.8 +/- 3.0 | <0.0001 |
| **Risk factors** | **Total mean** | **Medium Level** | **Vs** | **High Level** | **P=value** |
| Mean depth in BH-diving level | 22.2 +/- 8.5 | 16.1 +/-4.4 | Vs | 27.0+/-8.0 | <0.0001 |
| Max depth in BH-diving level | 33.2 +/- 8.2 | 27.41 +/- 5.5 | Vs | 38.8 +/-7.0 | <0.0001 |

C)

| **Risk factors** |  | |  |  |
| --- | --- | --- | --- | --- |
| **BMI total mean = 23.8 +/- 2.4** | **Above the mean** | **Below the mean** | | **P=value** |
| BMI in mean of depth | 23.8 +/-2.3 | 23.9 +/-2.5 | | p=0.94 |
| BMI in Maximum of depth | 23.8 +/-2.5 | 23.9 +/- 2.2 | | P=0.80 |
| BMI in N° of Dives | 23.9 +/- 2.5 | 23.7 +/- 2.2 | | P=0.73 |
| BMI in BH-diving level | 23.5+/-1.9 | 24.2 +/-2.8 | | P=0.29 |
|  |  |  | |  |
|  |  |  | |  |
| **AGE total mean = 43.2 +/- 9.8** | **Above the mean** | **Below the mean** | | **P=value** |
| AGE and mean of depth | 43.1 +/-10.1 | 47.5 +/-9.6 | | P=0.90 |
| AGE and Maximum of depth | 43.1 +/-10.3 | 42.5 +/-9.3 | | P=0.90 |
| AGE and N° of Dives | 43.7 +/- 8.8 | 42.7 +/- 11.0 | | P=0.73 |
| AGE and BH-diving level | 43.9+/-9.7 | 42.5 +/-10.1 | | P=0.62 |
|  |  |  | |  |
|  |  |  | |  |
| **Swimming Vs Sea** | **Sea** | **Swimming** | | **P=value** |
| BMI in environmental | 23.8 +/-2.5 | 23.8 +/- 2.5 | | P=0.99 |
| Mean depth in environmental | 18.3 +/-6.4 | 27.4 +/-8.5 | | <0.0001 |
| Max depth in environmental | 31.8 +/-8.7 | 35.0 +/-7.3 | | P= 0.30 |
