# Supplementary material for: Endothelial Nitric Oxide Production and Antioxidant Response in Breath-Hold Diving: Genetic Predisposition or Environment Related?
Source: Front Physiol. 2021 Jul 9;12:692204. doi: 10.3389/fphys.2021.692204 (PMC8300565; doi:10.3389/fphys.2021.692204)
Supplement: Supplementary file 2 [file Table_2.DOCX]

**Endothelial Nitric Oxide production and Antioxidant Response in BH Diving:**

**genetic predisposition or environment related?**

D. Cialoni^1,2,3^, A. Brizzolari^2,4^, M. Samaja^4^, G. Bosco^1^, M. Paganini^1^, M. Pieri^2^, N. Sponsiello^3,^ V. Lucchini^5^, V. Lancellotti^6^, and A. Marroni^2^

1) Environmental Physiology and Medicine Laboratory, Department of Biomedical Sciences,

University of Padua, Padua, Italy.

2) DAN Europe Research Division, Roseto degli Abruzzi, Italy

3) Apnea Academy Research, Padua, Italy

4) Department of Health Sciences, Università degli Studi di Milano, Milan, Italy

5) NGB Genetics Srl c/oUniversità di Ferrara - Bologna, Italy

6) Cardiothoracic and Vascular Department, Azienda Ospedaliero-Universitaria Pisana (AOUP), Pisa, Italy

Contact Information: Cialoni Danilo, DAN Europe, Contrada Padune 11, 64026 Roseto degli Abruzzi (TE), Italy. Phone: 39.085.8930333; Fax: 39.085.8930050; E-mail: dcialoni@daneurope.org

Keywords: Nitric oxide; Breath-Hold Diving; Diving; oxidative stress

Table II

No statistically significant changes were found in NOx in the different genetic variant investigated

| **Vascular related**  **Polymorphism** | **% of control value** | | | **P=Value** | | |
| --- | --- | --- | --- | --- | --- | --- |
|  | CC | CT | TT | CC Vs CT | CC Vs TT | CT Vs TT |
| ENOS_rs2070744 T30 | 59.7 +/17.5 | 74.7 +/-21.5 | 76.5 +/-43.3 | P= 0.58 | P= 0.58 | P= 0.85 |
| ENOS_rs2070744 T60 | 208.3 (28.4-278.8) | 108.1 (48-440.6) | 126.4 (24.6-251.9) | P> 0.99 | P> 0.99 | P> 0.99 |
|  |  |  |  |  |  |  |
|  | GG | GT | TT | GG Vs GT | GG vs TT | GT Vs TT |
| ENOS_rs1799983 T30 | 81.1 (1.81-194) | 65.3 (32.8-109.1) | 74.4 (59.3-80.1) | P=0.07 | P> 0.99 | P> 0.99 |
| ENOS_rs1799983 T60 | 126.4 (38.3-440.6) | 80.3 (24.6-362.1) | 228.8 (201.1-278.8) | P> 0.99 | P=0.44 | P=0.18 |
|  |  |  |  |  |  |  |
|  | DD | DI | II | DD Vs DI | DD vs II | DI Vs II |
| ACE T30 | 73.9 +/-21.4 | 76.0 +/-41.7 | 68.5 +/-27.1 | P=0.90 | P=0.90 | P=0.90 |
| ACE T60 | 128.3 (24.6-335.6) | 174.1 (28.4-440.6) | 71.9 (38.8-312.7) | P> 0.99 | P=0.73 | P=0.27 |

| **Antinfiammatory**  **Polymorphism** | **% of control value** | | | **P=Value** | | |
| --- | --- | --- | --- | --- | --- | --- |
|  | CC | CT | TT | CC Vs CT | CC Vs TT | CT Vs TT |
| IL1b T30 | 74.4 (31-112.8) | 69.1 (1.8-194) | 85.0 (49.5-110.5) | P> 0.99 | P> 0.99 | P= 0.62 |
| IL1b T60 | 88.0 (28.4-403.7) | 162.7 (24.6-440.6) | 126.4 (57.2-251.9) | P> 0.99 | P> 0.99 | P> 0.99 |
|  |  |  |  |  |  |  |
|  | CC | CT | TT | CC Vs CT | CC Vs TT | CT Vs TT |
| IL1RN T30 | 61.1 (1.81-110.5) | 70.2 (12.7-109.1) | 76.0 (31-194) | P> 0.99 | P=0.91 | P=0.69 |
| IL1RN T60 | 108.9 (75.7-121.7) | 101.8 (24.6-330.4) | 150.3 (28.4-440.6) | P> 0.99 | P> 0.99 | P=0.46 |
|  |  |  |  |  |  |  |
|  | GG | GC | CC | GG Vs GC | GG vs CC | GC Vs CC |
| TNFA T30 | 72.2 (1.8-194) | 76.9 (48.4-107.2) | N° Too small | P=0.90 | ----------- | --------- |
| TNFA T60 | 104.9 (24.6-440.6) | 172.4 (48-335.6) | N° Too small | P=0.77 | ----------- | ----------- |
|  |  |  |  |  |  |  |
|  | GG | GC | CC | GG Vs GC | GG vs CC | GC Vs CC |
| IL6 T30 | 67.8 (1.8-112.8) | 72.3 (12.7-194.0) | 76.0 (62.0-81.7) | P> 0.99 | P> 0.99 | P> 0.99 |
| IL6 T60 | 174.1 (28.4-403.7) | 104.9 (24.6-335.6) | 134.5 (50.4-440.6) | P= 0.42 | P> 0.99 | P> 0.99 |

| **Antioxidant**  **Polymorphism** | **% of control value** | | | **P=Value** | | |
| --- | --- | --- | --- | --- | --- | --- |
|  | CC | CT | TT | CC Vs CT | CC Vs TT | CT Vs TT |
| SOD2_ T30 | 61.3 (31.0-110.5) | 75.7 (1.8-194.0) | 76.5 (57.7-92.2) | P=0.48 | P> 0.99 | P> 0.99 |
| SOD2_ T60 | 132.6 (42.4-724.8) | 132.4 (24.6-403.7) | 91.9 (62.7-440.6) | P> 0.99 | P> 0.99 | P> 0.99 |
|  |  |  |  |  |  |  |
|  | GG | GA | AA | GG Vs GA | GG vs AA | GT Vs AA |
| CAT_rs1001179 T30 | 78.5 (31.0-194) | 72.7 (1.8.-112.8) | 49.5 (32.1-98.0) | P> 0.99 | P= 0.22 | P= 0.56 |
| CAT_rs1001179 T60 | 104.9 (24.6-335.6) | 163.4 (38.8-440.6) | 93.2 (46.7-724.8) | P= 0.18 | P> 0.99 | P=0.91 |
|  |  |  |  |  |  |  |
|  | GG | GA | AA | GG Vs GA | GG vs AA | GT Vs AA |
| PON1 T30 | 82.2 (32.1-107.2) | 71.8 (12.7-110.5) | 72.3 (1.8-194.0) | P= 0.90 | P=0.90 | P=0.53 |
| PON1 T60 | 62.7 (46.7-210.3) | 81.01 (24.6-724.8) | 151.5 (48.3-403.7) | P> 0.99 | P=0.34 | P=0.16 |
